# Retrotrigonal Layer Dissection from a Posterior Approach Enables Bladder Neck Preservation in Robot-Assisted Radical Prostatectomy

**DOI:** 10.3390/jcm13051258

**Published:** 2024-02-22

**Authors:** Satoshi Washino, Kimitoshi Saito, Yuhki Nakamura, Tomoaki Miyagawa

**Affiliations:** Department of Urology, Jichi Medical University Saitama Medical Center, Saitama 330-0834, Japansh2-miya@jichi.ac.jp (T.M.)

**Keywords:** retrotrigonal layer, robot-assisted radical prostatectomy, bladder neck dissection, bladder neck preservation

## Abstract

Background: We developed a novel surgical technique: dissection of the retrotrigonal layer from a posterior approach in robot-assisted radical prostatectomy (RARP). This approach enables earlier access to the posterior space during bladder neck dissection and helps preserve the bladder neck. We evaluated the safety and efficacy of this technique in terms of bladder neck preservation Methods: We retrospectively reviewed 238 consecutive patients who underwent RARP using this technique from August 2021 to September 2023. Key metrics included the success rate of accessing the posterior space prior to bladder neck opening during the dissection and the rate of bladder neck preservation. In addition, oncological and surgical safety, as well as continence recovery, were assessed. Results: The median age and prostate-specific antigen level were 72 (65–75) years and 7.35 (5.40–11.5) ng/mL, respectively. In 153 (64%) patients, the posterior space was successfully accessed before bladder neck opening, with the success rate increasing from 56% in the series’ first half to 72% in the second half (*p* = 0.015). The bladder neck was preserved in 120 (53%) patients, and this rate increased from 39% in the first half to 63% in the second half (*p* = 0.0004). Positive surgical margins at the junction between the prostate and bladder were observed in 10 cases (4%). Bladder neck preservation correlated with early continence recovery (hazard ratio 1.37 [95% confidence interval 1.03–1.83], *p* = 0.030). The grade 3 complication associated with this technique occurred in one case (0.5%). Conclusion: Retrotrigonal layer dissection from a posterior approach in RARP enhances the safety and ease of bladder neck dissection and aids in its preservation, potentially leading to improved continence recovery.

## 1. Introduction

Robot-assisted radical prostatectomy (RARP) was initially performed in 2000 by Binder et al. in Frankfurt, Germany [1], and by Abbou et al. in Creteil, France [2]. Since its inception, several modifications and refinements of surgical techniques have been introduced to optimize functional recovery post-RARP. These include the posterior approach [3], bladder neck preservation [4], and the Retzius-sparing technique [5].

One of the most challenging aspects of RARP is the dissection between the bladder and prostate. This complexity is accentuated in men with large prostates, pronounced median lobes, or prior transurethral surgery. The retrotrigonal layer, also termed vesicoprostatic muscles, consists of longitudinal muscles linking the bladder and prostate. This layer delineates the detrusor muscles from the seminal vesicles and deferens vasa. It serves a pivotal role in RARP by offering a landmark to pinpoint the posterior plane of prostatic dissection and also acts as the posterior layer for subsequent reconstruction. Misidentification or improper dissection of this layer can lead to bladder injury or misdissection of the prostate.

The posterior approach facilitates direct dissection of the seminal vesicles [6] and has been correlated with reduced operative durations in patients with larger prostate glands, as opposed to the anterior approach [3]. Following the isolation of seminal vesicles, the retrotrigonal layer becomes visible from the posterior approach. Its transection, when approached from the posterior, enables the anterior separation of the bladder from the prostate. This step is vital in the Retzius-sparing technique. We have integrated this technique into posterior–anterior RARP, where seminal vesicle isolation is performed from the posterior approach, followed by prostatectomy from the anterior perspective. This novel approach permits access to the posterior space prior to opening the bladder neck during its dissection, potentially making the dissection more straightforward and safer, and aiding in bladder neck preservation, which has been linked with enhanced continence recovery [7].

To date, no reports have been published on the dissection of the retrotrigonal layer from a posterior approach in posterior–anterior RARP. We conducted a retrospective review of patients treated with this innovative technique to assess its safety and effectiveness in bladder neck preservation.

## 2. Materials and Methods

This retrospective observational study received approval from the Institutional Review Board of Jichi Medical University, Saitama Medical Center (RinS20–058, 10 October 2022). We retrospectively analyzed 238 consecutive patients who underwent RARP using the novel technique of retrotrigonal layer dissection from the posterior approach between August 2021 and September 2023. Seven surgeons with varying experience with robotic surgery, ranging from novices to those who had handled > 200 cases, performed RARP using either the da Vinci Si^®^ Surgical System or the Xi^®^ Surgical System (Intuitive Surgical, Sunnyvale, CA, USA).

### 2.1. Surgical Techniques

#### 2.1.1. Posterior Approach: Isolation of Seminal Vesicle and Dissection of Deferens Vasa

The parietal peritoneum on the anterior surface of the Douglas space is incised. Then, the seminal vesicles and deferens vasa are isolated and sectioned.

#### 2.1.2. Dissection of the Retrotrigonal Layer from the Posterior Approach

**Figure 1:**
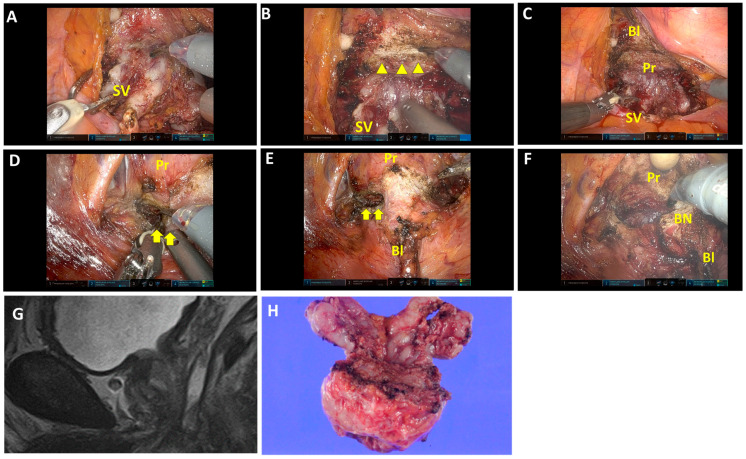
Surgical techniques of the retrotrigonal layer dissection from the posterior approach and bladder neck preservation. (**A**) Isolation of the seminal vesicle and dissection of deferens vasa in the posterior approach. (**B**) The retrotrigonal layer, pinkish longitudinal tissue, is transected (triangles). (**C**) The view after transection of the retrotrigonal layer. The bladder is separated anteriorly from the prostate. (**D**,**E**) Left lateral approach in bladder neck dissection. The posterior space is opened prior to the bladder neck opening (arrows). Detrusor muscles are detached from the prostate. (**F**) The bladder neck is preserved. (**G**,**H**) Magnetic resonance imaging (MRI) sagittal view and macroscopic findings of the prostate and seminal vesicle. SV, seminal vesicles; Bl, bladder; Pr, prostate; BN, bladder neck.

**Figure 2:**
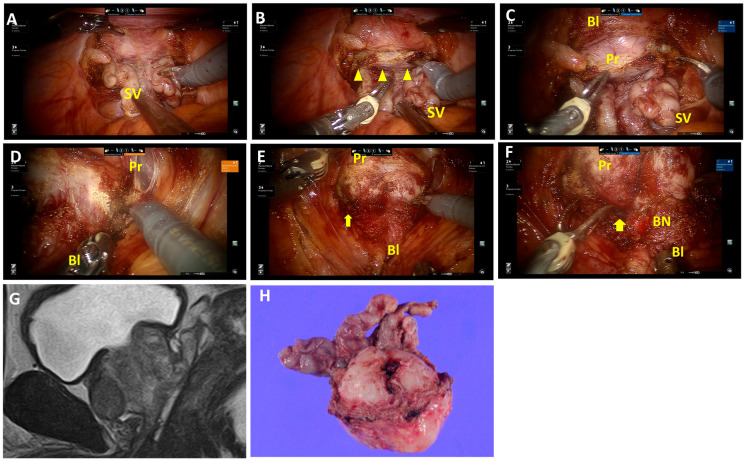
Surgical techniques of the retrotrigonal layer dissection from the posterior approach and bladder neck preservation in patients with an enlarged prostate. (**A**) Isolation of the seminal vesicle and dissection of deferens vasa in a posterior approach. (**B**) The retrotrigonal layer, pinkish longitudinal tissue, is transected (triangles). (**C**) The surface of the prostatic pseudocapsule can easily be identified after transection. This layer can be developed anteriorly. (**D**) Right lateral approach in bladder neck dissection. (**E**) Detrusor muscles are detached from the prostate. The posterior space is visible laterally (arrows). (**F**) The bladder neck is preserved (arrows). (**G**,**H**) MRI sagittal view and macroscopic findings of the prostate and seminal vesicle. Enlargement of the prostate gland is visible and the surface of the prostatic pseudocapsule at the protrusion site is exposed (**H**). SV, seminal vesicles; Bl, bladder; Pr, prostate; BN, bladder neck.

Fat tissues and vessels overlap the retrotrigonal layer (Figure 1A; Video S1) and are abundant on the lateral sides. Fat tissues and vessels, followed by the retrotrigonal layer, are transected approximately 0.5–1.0 cm above the seminal vesicle and deferens vasa. This allows the bladder wall to be separated anteriorly from the prostate (Figure 1B,C). In cases with an enlarged prostate or prominent median lobes, the retrotrigonal layer tends to be thinner, and the surface of the prostatic pseudocapsule can easily be identified after transection (Figure 2A,B; Video S2). Then, this layer can be developed anteriorly (Figure 2C). For nerve-sparing procedures, the lateral transection of the retrotrigonal layer and/or overlapping fat tissues and vessels are preserved.

#### 2.1.3. Development of the Retropubic Space

An inverted U-shaped incision is made on the parietal peritoneum to reveal the bladder and the anterior prostate. This is followed by exposure of the puboprostatic ligaments.

#### 2.1.4. Dissection of Endopelvic Fascia

The endopelvic fascia is bluntly dissected to expose the lateral surface of the prostate.

#### 2.1.5. Bladder Neck Preservation

The detrusor apron at the bladder neck is dissected and developed laterally (Figure 1D and Figure 2D). Then, the posterior space is opened laterally prior to the bladder neck opening (Figure 1D). Carefully, detrusor muscles are detached from the prostate, revealing the longitudinal urethral muscles in the center (Figure 1E). Then, the anterior urethral muscles are dissected to open the bladder neck, followed by the posterior bladder neck. The bladder neck is preserved (Figure 1F and Figure 2F). This procedure was consistently applied, except in cases where the prostate tumor was located near the proximal edge, adjacent to the bladder neck.

### 2.2. Objectives of the Study

In our cohort, we evaluated the following:

1. The success rate of opening the posterior space prior to opening the bladder neck during bladder neck dissection from an anterior perspective, as shown in Figure 1D,E.
2. The success rate of bladder neck preservation. Bladder neck preservation was defined as dissecting anterior urethral muscles and the opening of the bladder neck being less than the width of a 16 French Gauge Foley catheter (5.3 mm) following its dissection.
3. Rate of complications and positive surgical margins (PSMs).
4. Continence recovery. Continence was defined as the use of zero or one pad for safety daily following urethral catheter removal.

In addition, we compared results for objectives 1 and 2 between the first and second halves of our cohort series and between cases performed by less-experienced surgeons (experiencing <40 cases) and experienced surgeons (with ≥40 cases) to determine if there was a learning curve. A total of 44 (18%) cases were performed by less-experienced surgeons. We also contrasted continence recoveries between patients who achieved bladder neck preservation and those who did not.

### 2.3. Statistical Analysis

Data are presented as median and interquartile range (IQR) unless otherwise specified. Fisher’s exact test and one-way ANOVA with Tukey’s multiple comparisons test were used to compare variables. Continence recovery rates were analyzed with the Kaplan–Meier method and the log-rank test. All statistical evaluations were conducted using GraphPad Prism software version 9.0 (GraphPad Software, La Jolla, CA, USA).

## 3. Results

### 3.1. Patients’ Background and Pathological Characteristics

The median age and prostate-specific antigen level were 72 (65–75) years and 7.35 (5.40–11.5) ng/mL, respectively (Table 1). National Comprehensive Cancer Research (NCCN) risk was categorized as low or less in 8 patients, intermediate favorable in 86, intermediate unfavorable in 47, high in 73, and very high in 21, with 3 patients not assessed. A total of 227 patients underwent prostate magnetic resonance imaging (MRI), with a median MRI-determined prostate volume of 34.4 (26.2–44.5) cm^3^. The prostate protrusion was noted in 55 of these 227 patients, with a median length of 9.0 (6.80–11.1) mm from the bladder neck. The median follow-up period was 11.8 (5.90–12.1) months.

Neoadjuvant androgen deprivation therapy was administered to 25 patients, and pathological analysis was available for the remaining 213 patients. The proportion of patients with pT2, pT3a, and ≥pT3b was 69%, 24%, and 7%, respectively, whereas that of those with ≥Grade Group 4 was 24% (Table 2).

### 3.2. Surgical Outcomes

A total of 47 patients underwent nerve sparing. The median console time and blood loss were 178 (155–210) min and 30 (19–50) mL, respectively.

**Figure 3:**
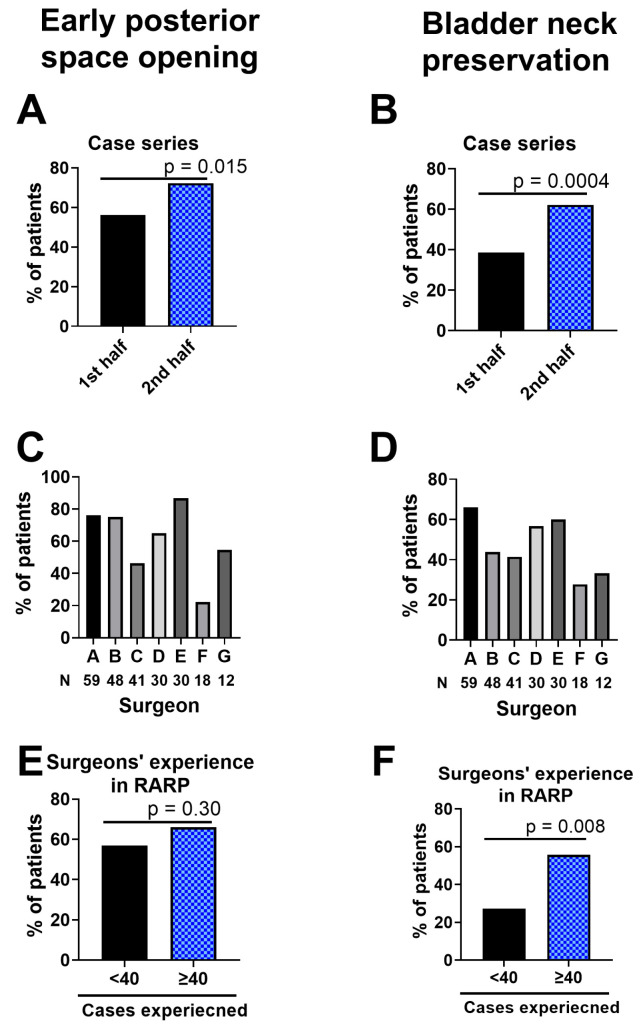
Success rate of the early posterior space opening and bladder neck preservation. The success rate of early posterior space opening (**A**) and bladder neck preservation (**B**) was higher in the second half of the series compared with the first half of the series, while the rates varied among surgeons (**C**,**D**) and were higher in cases performed by experienced surgeons (**E**,**F**).

In 153 (64%) patients, the posterior space was successfully opened prior to the bladder neck opening from the anterior perspective. The success rate increased from 56% (67 out of 119) in the first half of the series to 72% (86 out of 119) in the latter half (*p* = 0.015) (Figure 3A). In 120 (53%) patients, the bladder neck was successfully preserved. The success rate for bladder neck preservation grew from 39% (46 out of 119) in the initial series half to 63% (74 out of 119) in the latter half (*p* = 0.0004) (Figure 3B). The success rate in the early posterior opening and bladder neck preservation depended on the surgeons (up to 87% in the early posterior opening; 66% in the bladder neck preservation) and their experience in RARP (Figure 3C–F). A positive association was evident between the success in bladder neck preservation and the prior opening of the posterior space (61% [94 out of 153 in the prior opening of the posterior space (+)] vs. 30% [26 out of 85 in the prior opening of the posterior space (–)], *p* < 0.0001). Conversely, both large prostate volume and prostate protrusion were negatively associated with bladder neck preservation success (33% [13 out of 39] in prostate volume ≥ 50 mL vs. 54% [101 out of 188] in prostate volume < 50 mL, *p* = 0.023; 40% [22 out of 55] in protrusion (+) vs. 54% [92 out of 172] in protrusion (–), *p* = 0.09). Among the 63 patients with a large prostate (volume ≥ 50 cm^3^) and/or positive prostate protrusion, 7 (11%) needed bladder neck reconstruction.

Cases successfully preserving the bladder neck were associated with a shorter console time compared to cases without bladder neck preservation or requiring bladder neck reconstruction (170 [150–198] vs. 182 [158–213] or 203 [170–243] min, *p* = 0.15 or *p* = 0.0011).

### 3.3. Complications

There was one case (0.5%) of grade 3 complication associated with this technique who experienced bladder injury during retrotrigonal layer dissection, necessitating wall closure. MRI in this case revealed a very thin posterior bladder wall. No patients experienced misdissection into the prostate or ureteral injury.

### 3.4. Positive Surgical Margins

Among the 213 patients who did not receive neoadjuvant androgen deprivation therapy, PSMs were noted in 53 (25%) patients (Table 2). Specifically, PSMs were observed in 21 of 146 patients (14%) with pT2 and 32 of 67 (48%) with pT3a or higher. Ten cases (4%) had PSMs at the juncture between the prostate and bladder, whereas none of them experienced biochemical recurrence within a median follow-up of 5.9 months.

### 3.5. Continence Recovery

**Figure 4:**
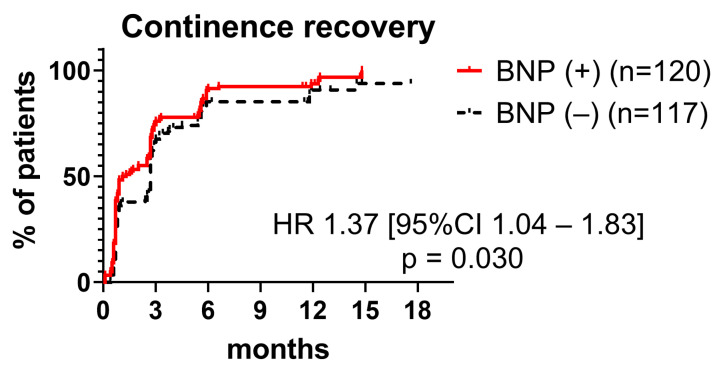
Continence recovery in cases with (red line) and without bladder neck preservation (black dashed line). Continence recovery was superior in cases with bladder neck preservation compared to those without. BNP, bladder neck preservation; HR, hazard ratio; CI, confidence interval.

Post-RARP, the continence rates stood at 42%, 72%, and 92% at 1, 3, and 12 months, respectively. The preservation of the bladder neck was correlated with early continence recovery (hazard ratio [HR] 1.37 [95% confidence interval (CI) 1.03–1.83], *p* = 0.030; 48% vs. 36% at 1 month; 76% vs. 68% at 3 months; 94% vs. 91% at 12 months) (Figure 4).

## 4. Discussion

We introduced a novel surgical technique—dissecting the retrotrigonal layer from a posterior approach in posterior-anterior RARP—and demonstrated its utility for safely performing bladder neck dissections and facilitating bladder neck preservation.

### 4.1. Dissection of the Retrotrigonal Layer and Bladder Neck Preservation

The retrotrigonal layer is characterized as a pinkish-white midline strip with vertically oriented fibers that extend from the bladder trigone (anteriorly) to the base of the prostate (inferiorly) [8]. Laterally, this layer stretches to the proximal neurovascular pedicles and the effacing detrusor fibers on the prostatic capsule. Identifying the correct posterior plane following the transection of the bladder neck can be challenging, particularly without a preliminary seminal vesicle dissection. Erroneous dissection might result in capsular incision of the prostate or inadvertent cystotomy of the posterior bladder wall. In the traditional anterior approach, dissecting beyond the retrotrigonal layer exposes the posterior space [9]. With our innovative technique, the posterior space can be accessed earlier from the anterior side. This not only streamlines the bladder neck dissection but also simplifies its preservation. Our findings indicate that the success rate of preservation is positively correlated with the preliminary opening of the posterior space before the bladder neck. This success rate also showed an upward trend in the latter half of our series and varied among surgeons (Figure 3), suggesting a learning curve. Accomplishment of bladder neck preservation was associated with a shorter operative time compared to the non-bladder neck preservation group or the bladder neck reconstruction group in the present study, which might be one of the advantages of bladder neck preservation. It is important to note that thermally dissecting the lateral tissues of the retrotrigonal layer might damage the neurovascular pedicles, which should remain intact in cases undergoing nerve sparing. The retrotrigonal layer is the key structure for posterior reconstruction following prostatectomy [10]. Appropriate dissection of the retrotrigonal layer could help the reconstruction, potentially leading to improved continence recovery.

### 4.2. Cases with Large Prostate Glands and Median Lobes

It has been shown that dissecting seminal vesicles from a posterior approach is associated with a reduced operative time in patients with larger prostate glands compared to an anterior approach [3]. Our new technique is a refinement of the traditional posterior approach. In instances with large prostate glands or a significant median lobe, the detrusor muscles remain well-preserved (Figure 2F), which makes it unnecessary to extensively open the bladder neck. Bladder neck reconstruction was only needed in 11% of patients with a large prostate gland and/or prostate protrusion in the present study.

### 4.3. PSMs and Safety

The PSM rate was 25% across all cohorts, 14% in pT2, and 48% in pT3 or higher. These figures align closely with findings from previous studies [11,12]. While bladder neck preservation might elevate the risk for PSMs, a meta-analysis determined no association between bladder neck preservation and the overall PSM rate or PSM at the prostate base [7]. In this study, a mere 4% of patients had PSMs at the junction between the prostate and bladder, which aligns closely with findings from previous studies (5.7 to 6.8%) [13], and none of them experienced an early biochemical recurrence. These suggest that the procedure is likely safe concerning oncological outcomes when selection criteria are judiciously applied.

In the present study, no patients experienced misdissection into the prostate or ureteral injury despite 18% of RARPs being performed by less-experienced surgeons, suggesting that this procedure may enhance the safety of bladder neck dissection. However, bladder wall injury during retrotrigonal layer dissection occurred in one (0.5%) patient who exhibited a very thin bladder wall by MRI. Dissection of the retrotrigonal layer should be performed carefully in such patients.

### 4.4. Continence Recovery

The bladder neck preservation technique is one of the methods introduced to facilitate urinary continence through the sparing of the internal sphincter by isolating and dissecting the prostatic urethra [13,14,15,16]. In contrast, the effect of bladder neck preservation on urinary continence has been controversial [17,18]. Meta-analyses have shown that preserving the bladder neck expedites the return of urinary continence post-RARP, with an odds ratio (OR) of 2.88 at 3–4 months and OR 3.23 at 24 months [7]. Our findings resonate with these results, indicating that bladder neck preservation is associated with positive continence recovery (Figure 4).

### 4.5. Limitations

This study, a retrospective case series, did not juxtapose its outcomes with the traditional anterior approach. Several surgeons with varying degrees of expertise performed the RARP, which may have led to uneven results and a longer learning curve in terms of the success of bladder neck preservation. Additionally, assessment of erectile function is missing in the present study.

## 5. Conclusions

Transecting the retrotrigonal layer from a posterior approach in posterior–anterior RARP not only simplifies and secures bladder neck dissection but also facilitates bladder neck preservation.

## Figures and Tables

**Table 1:** Patient background (n = 238).

Variables	Median (IQR)
Age, years	72 (65–75)
Body mass index	23.9 (21.9–25.1)
Serum PSA, ng/mL	7.35 (5.40–11.5)
NCCN risk classification, n (%)	
Low or less	8 (3)
Intermediate favorable	86 (36)
Intermediate unfavorable	47 (20)
High	73 (31)
Very high	21 (9)
Not assessed	3 (1)
MRI prostate volume (cm^3^)	34.4 (26.9–45.4)
<50.0, n (%)	188/227 (83)
50.0–79.9, n (%)	33/227 (15)
≥80.0, n (%)	6/227 (3)
Prostate protrusion, n (%)	55/227 (24)

PSA, prostate-specific antigen; NCCN, National Comprehensive Cancer Research; MRI, magnetic resonance imaging; IQR, interquartile range.

**Table 2:** Pathological characteristics (n = 213).

Variables		n (%)
Pathological T stage	pT2	146 (69)
	pT3a	52 (24)
	≥pT3b	15 (7)
Grade group	1	3 (1)
	2	96 (45)
	3	62 (29)
	4	17 (8)
	5	35 (16)
Positive surgical margin	Total	53 (25)
	pT2	21/146 (14%)
	≥pT3a	32/67 (48%)

## Data Availability

The data are available on request by contacting the corresponding author (S.W.).

## Supplementary Materials

The following supporting information can be downloaded at: https://www.mdpi.com/article/10.3390/jcm13051258/s1; Video S1: Surgical techniques of the retrotrigonal layer dissection from the posterior approach and bladder neck preservation in a case without prostate enlargement or protrusion; Video S2: Surgical techniques of the retrotrigonal layer dissection from the posterior approach and bladder neck preservation in a case with prostate enlargement.
